# Biodiversity within *Melissa officinalis*: Variability of Bioactive Compounds in a Cultivated Collection

**DOI:** 10.3390/molecules23020294

**Published:** 2018-01-31

**Authors:** Remigius Chizzola, Ulrike Lohwasser, Chlodwig Franz

**Affiliations:** 1 Institute of Animal Nutrition and Functional Plant Compounds, University of Veterinary Medicine Vienna, Veterinaerplatz 1, 1210 Vienna, Austria; Chlodwig.Franz@vetmeduni.ac.at; 2 Leibniz Institute of Plant Genetics and Crop Research (IPK), Corrensstraße 3, Seeland, OT 06466 Gatersleben, Germany; lohwasser@ipk-gatersleben.de

**Keywords:** Lamiaceae, *Melissa officinalis*, lemon balm, essential oil, polyphenols, rosmarinic acid

## Abstract

Phytochemical characters were evaluated in a five-year-old lemon balm collection consisting of 15 and 13 subspecies *officinalis* and *altissima* accessions, respectively. Stems were lower in essential oil than leaves. First cut leaves (June) gave more oil than those of the second cut (August). Subspecies *officinalis* plants had leaf oils rich in geranial, neral and citronellal in various proportions in the first cut. However, in the second cut the oils from all accessions appeared very similar with 80–90% geranial plus neral. Leaf oils of subsp. *altissima* contained sesquiterpenes (β-caryophyllene, caryophyllene oxide, germacrene D) and also further monoterpenes in the second cut. Leaves had higher rosmarinic acid (RA) contents than stems. More RA was in subsp. *officinalis* than subsp. *altissima* leaves. First cut leaves were richer in RA than those from second cut. Total phenolics and antioxidant parameters showed that lemon balm is a valuable source of plant antioxidants.

## 1. Introduction

Lemon balm has a long tradition of use as a spice, medicinal plant and herbal tea with mild sedative properties. It originates from the eastern Mediterranean and western Asia region and is nowadays naturalized and cultivated in many countries [1,2]. In Europe, two subspecies identified as *Melissa officinalis* L. (syn. *M. officinalis* subsp. *officinalis*) and *Melissa officinalis* subsp. *altissima* (Sm.) Arcang., Lamiaceae (according to ThePlantList.org) were differentiated, that can be distinguished by the indumentum of the leaves and the shape of the middle tooth of the upper lip of the fruiting calyx [1]. In the following they will be denoted as MOFF and MALT, respectively. In phytotherapy internal uses include therapeutic indications such as restlessness and irritability, as well as symptomatic treatment of digestive disorders. External uses are treatments of herpes labialis [2,3]. In traditional medicine a wide range of applications is documented against various illnesses ranging from headache, migraine, to digestion problems and nausea, to insomnia, anxiety, vertigo and syncope, but also anaemia, asthma, bronchitis, amenorrhea, heart failure, arrhythmias, epilepsy, and rheumatisms have been addressed [4]. Modern pharmacological studies demonstrate that *M. officinalis* has several biological activities including antioxidant, hypoglycemic, hypolipidemic, antimicrobial, anticancer, antidepressant, anxiolytic, antinociceptive, anti-inflammatory and spasmolytic properties [5,6,7]. Special attention should be given to antiviral effects [8]. Also, the use in the food industry is of interest due to the antimicrobial and antioxidant properties of the plant [5].

The plant contains low amounts of essential oils that include geraniol, citronellal, geranial and neral as citrus aroma compounds and sesquiterpenes, mainly β-caryophyllenene and caryophyllene oxide in varying proportions. Phenolic compounds present in the plant are hydroxycinnamic acid derivatives such as rosmarinic acid and flavonoids. Triterpenoids are also reported [2,3].

The purposes of this study were: (i) to investigate the variability in essential oil composition from different accessions of balm grown under Central European climate conditions where two harvests of the preflowering herb are possible and (ii) to study the variation in rosmarinic acid and antioxidant activity in extracts occurring at the same time.

The findings may support the further exploitation of lemon balm as a source of natural antioxidants with high rosmarinic acid contents. Additionally, highlighting plant sources producing essential oils high in the mentioned citrus aroma monoterpenes might be of interest for various further uses of lemon balm.

## 2. Results and Discussion

### 2.1. Morphological Characters

Altogether, 28 accessions were evaluated, 15 belonging to MOFF (*M1*, *M2*, *M4–M8*, *M10*, *M11*, *M13*, *M16*, *M25–M27*, *M29*) and 13 to MALT (*M9*, *M12*, *M14*, *M15*, *M17–M24*, *M28*). Accession *M3* had to be excluded from the study as no plant material was available. The variability of simple morphological traits as shoot development and leaf sizes has been recorded in the second year of growth and both subspecies were compared as presented in Table 1. Significant differences were found for the characters shoot length (*p* = 0.002), distance of nodes (*p* < 0.001) and the ratio leaf length/width (*p* = 0.015). Thus, on average plants of MALT had longer shoots and spaces between nodes but a lower leaf length to width ratio, and their leaves appeared more orbicular.

### 2.2. Essential Oil Yield

The essential oils were isolated by hydrodistillation and recovered in hexane. Some samples very low in oil did not allow a direct reading of the oil content in the distillation apparatus. Therefore, the oil content of all samples was calculated from the total area of all peaks in Gas chromatography/flame ionization detection (GC/FID) assuming the same response as for the internal standard cyclododecanone (Table 2). The oil content of the leaves proved to be very variable: Plants of MOFF had higher oil content than those of MALT (*p* = 0.004 and *p* < 0.001 for the first and second cut, respectively). The exact values are provided in Table S1 in the Supporting Information. At the same time leaves contained more essential oil than stems. Furthermore, crop management appeared to have an important influence on essential oil accumulation as the regrowth harvested at the second cut had considerably higher oil contents as compared to the first cut. During plant development the highest oil contents occur with around 0.4% in the herb during the flowering stage, while large variation may occur from one year to another [9]. In the present study the highest oil contents were recorded in some MOFF leaves (*M4*, *M8*, *M25*, *M26*, 8955–12,669 µg/g) of the second cut. There is also the observation that the oil content increased with the year of cultivation [10].

### 2.3. Essential Oil Composition

In leaves and stems 65 and 43 essential oil compounds could be identified, respectively. The detailed listing is available with the Supporting Information Tables S2 and S3. Bar charts showing the main oil compounds of the accessions are given in Figures S1 and S2 in the Supporting Information. To study the complex oil patterns in the leaves a multivariate approach has been attempted using hierarchical cluster analysis (HCA) and principal component analysis (PCA) with selected essential oil compounds as variables and the accessions as cases. In PCA also rosmarinic acid, total phenolics and antioxidant activity of both cuts have been included as variables.

**Figure 1:**
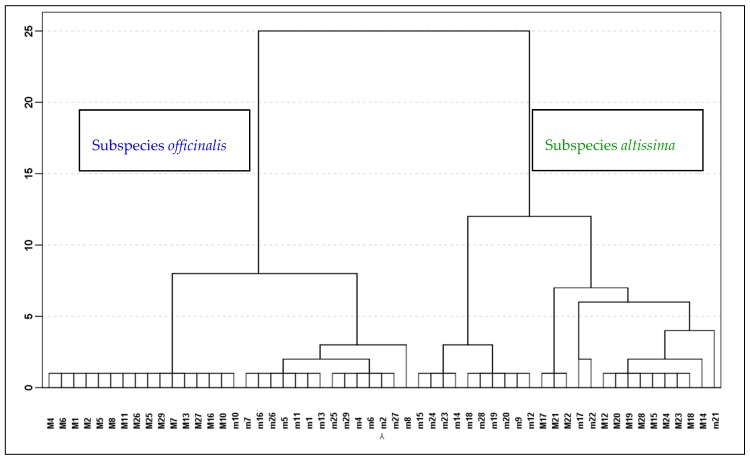
Dendrogram showing the similarities between the leaf oils of the accessions of *Melissa offcinalis*. (m1–m29: samples of the first cut, M1–M29: samples of the second cut).

**Figure 2:**
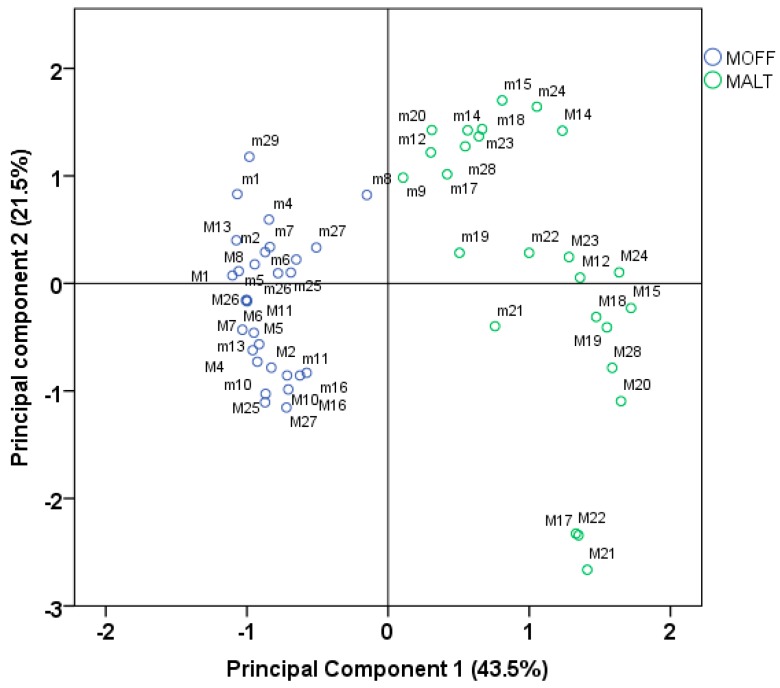
Projection of *Melissa officinalis* accessions onto the plane of two main PCA factors obtained for contents % essential leaf oil, leaf rosmarinic acid and antioxidant parameters. (m1–m29: samples of the first cut, M1–M29: samples of the second cut).

**Figure 3:**
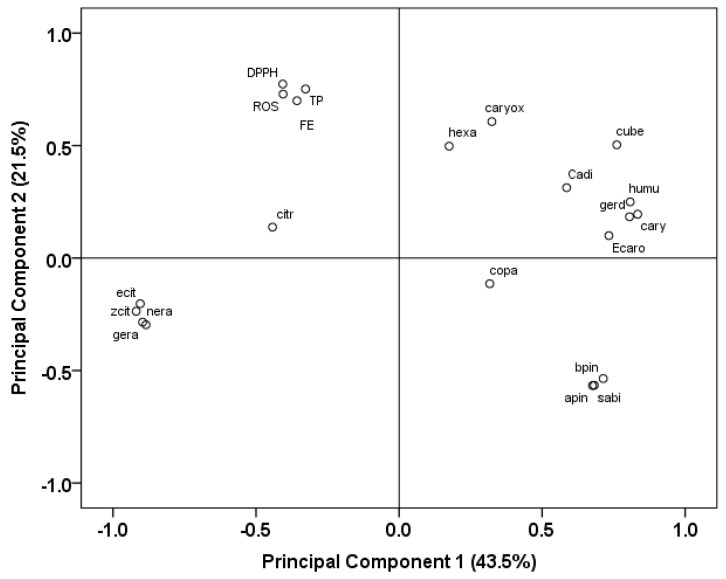
Loading of the leaf essential oil compounds, rosmarinic acid and antioxidant parameters on the components in PCA. The analysis included as variables: apin: α-pinene, bpin: β-pinene, cadi1: α-cadinol, cary: β-caryophyllene, caryox: caryophyllene oxide, citr: citronellal, copa: α-copaene, cube: β-cubebene, DPPH: DPPH antioxidant activity, Ecaro: 9-*epi-E*-Caryophyllene, ecit: *E*-isocitral, FE: FRAP, gera: geranial, gerd: germacrene D, hexa: hexadecanoic acid, humu: α-humulene, nera: neral, ROS: rosmarinic acid, sabi: sabinene, TP: total phenolics, zcit: Z-isocitral.

The dendrogram from HCA (Figure 1) presents two distinct main clusters, the left containing all accessions of MOFF while the right grouped the samples MALT. Plants of MOFF had the monoterpene aldehydes geranial (=*E*-citral, citral a) and neral (=*Z*-citral, citral b) with citrus-like aroma as major oil compounds, while in MALT the sesquiterpenes β-caryophyllene, caryophyllene oxide and germacrene D were characteristic compounds. The oils of the MALT leaves contained also appreciable amounts of the monoterpenes α-pinene, β-pinene and sabinene. Starting with 21 variables, the PCA calculated four components having eigenvalues greater than one and representing together 83.9% of total variance. The first axis accounted for 43.5% and the second for 21.5% of the variance (Figure 2 and Figure 3). The scoring plot of the first two components could also clearly differentiate between the two subspecies (Figure 2). MOFF samples formed a group with negative factor 1 scores, while all MALT accessions had positive factor 1 scores.

The formation of subclusters in HCA (Figure 1) and the division of the scores in PCA (Figure 2) show for MOFF samples a lower variability than for MALT samples. In addition, in both subspecies, the oil compositions changed noticeably from the first to the second cut. In PCA first cut samples were mostly associated with a positives component 2 score while the second cut samples had rather a respective negative score (Figure 2).

In the dendrogram the first and the second cut samples of MOFF were classed into two distinct subclusters. The plants from the first cut had geranial as main compound, varying amounts of neral and citronellal and around 9% caryophyllene oxide in their oils. To compare, an oil from cultivated blooming plants of the Balkans with 23.4% geranial, 16.5% neral and 13.7% citronellal showed a similar composition as the present first cut oils [11] and similar oils were also reported from blooming Slovakian plants [12]. However, the oils from the present study second cut appeared more homogeneous: in each of 15 accessions geranial and neral accounted together for 80–90% of the oil and the variability between the accessions of these two compounds with coefficients of variation (CV%) of 5.1% and 4.2%, respectively, was remarkably low. A similar result was obtained by Adzet et al. (1992) [13] where in 25 biotypes of lemon balm cultivated in the Spanish Ebro region the sum geranial + neral made up 93–96% of the respective oils with CV% less than 7%. In contrast to our results, in this latter study the ratio of these two compounds varied little between July/August and November [10]. Accession *M10* had already in the first cut oil very high geranial and neral levels like the oils from the second cut and was therefore in the subcluster of the second cut samples. In comparison with other MOFF samples the first cut from *M8* had the lowest geranial and neral and the highest β-caryophyllene and germacrene D percentages and had in consequence its distinct position in the dendrogram and on the score plot.

With exception of the accessions *M17*, *M21* and *M22*, also MALT samples of the first and the second cut could well be separated in distinct sublclusters (Figure 1). 

To summarize, the individual oil compounds loadings on the principal components are represented in Figure 3. The loading on component 1 shows as mentioned above a strong differentiation of the citrus-like aroma monoterpenes from both cuts (citronellal, neral, geranial) typical for MOFF oils from the sesquiterpenes that are characteristic for MALT. Here these monoterpenes were strongly associated with negative values while the mentioned sesquiterpenes loaded with positive values. Further monoterpenes α-pinene, β-pinene and sabinene which were conspicuous in MALT leaves of the second cut had positive loadings on component 1 and negative loadings on component 2. These three monoterpenes, almost absent in MOFF, were present in low amounts in several first cut MALT samples. Of the latter, only accession *M21* had at this time 12.6% sabinene, 10.8% β-pinene and 4.5% α-pinene. However, in the second cut the accessions *M17*, *M21* and *M22* were rich in these three compounds that together made up 55–60% of the respective oils. In consequence, they formed a subgroup with negative factor 2 scores in PCA and a distinct subcluster in HCA.

Hexadecanoic acid, present mainly in MALT first cut samples (up to 5.9%) and having positive component 2 scores, loaded accordingly positively on principal component 2. Furthermore *epi*-caryophyllene could be detected in MALT plants of the second cut, while hexadecanoic acid was not found in these samples. A subcluster in HCA with five accessions (*M19*, *M28*, *M18*, *M12* and *M20*) had the highest caryophyllene oxide contents in the oils of the first cut.

In the present MOFF leaf oils geranial and neral were positively correlated (*R* = 0.976, *p* < 0.001) but negatively correlated with citronellal (*R* = −0.920 and −0.935, *p* < 0.001). Additionally, geranial and neral were negatively correlated with caryophyllene oxide, germacrene D and β-caryophyllene while caryophyllene oxide showed a positive correlation with citronellal and germacrene D. In MALT leaf oils α-pinene, β-pinene and sabinene were strongly correlated (*R* = 0.993–0.998, *p* < 0.001) while caryophylene oxide was negatively correlated with these three monoterpenes (*R* = −0.728 to −0.756, *p* < 0.001) and with germacrene D (*R* = −0.521, *p* = 0.008) and β-caryophyllenene (*R* = −0.439, *p* = 0.028). Small amounts of the sesquiterpenes α-copaene, β-cubebene and cadinol occured in samples from both subspecies and were usually higher in MALT.

Stem essential oils were analyzed from five selected accessions of each subspecies (Supporting Information, Figures S3 and S4). Like in the leaves, the stem oils of the first cut MOFF plants had geranial, neral and citronellal in varying proportions but not more than 35% together, so these oils had a high proportion of sesquiterpenes such as β-caryophyllene, caryophyllene oxide and α-copaene. The latter reached 18% of the oil in accession *M10*. In the second cut, the stems had as the leaves geranial and neral as main oil compounds, ranging together between 40% and 85% of the respective oils. In contrast, germacrene D that played only a marginal role in MOFF stems was a major compound in MALT stem oils. In the oil from the first cut of accession *M21* it was the only detectable compound. Other sesquiterpenes present were caryophyllene oxide and β-caryophyllene and in accessions *M12* and *M20* also α-copaene. Stems of the first cut had more caryophyllene oxide and less β-caryophyllene than the respective stems of the second cut. The accessions *M17*, *M21* and *M22*, having high proportions of β-pinene, sabinene and α-pinene in the leaves, had these compounds also in their stem oils from the second cut. By this way the stem oils reported here differed clearly from a stem oil of Iranian plants where the main constituents were: *n*-hexadecanoic acid (47.4%), (*Z,Z*)-9,12-octadecadienoic acid (14.9%), dodecanoic acid (4.6%), β-caryophyllene (4.2%) and geraniol (2.2%) [14].

In sum, lemon balm essential oils show a great variability and plasticity. Literature references in most cases do not differentiate into the two subspecies when referring to essential oil composition but a great variation is documented. There are reports of oils having geranial and neral as main compounds. Leaf oil from plants grown in Algeria, being composed of 44.2% geranial, 30.2% neral, 6.3% citronellal and less than 4% sesquiterpenes, was similar to the present leaf oils from the second cut [15]. Further citrus-like aroma monoterpenes may also play a major role in the oils. Several Turkish lemon balm oils had citronellol (37–44%) as main compound [16]. An Iranian lemon balm flower essential oil displayed *trans*-carveol (28.9%), citronellol (25.2%), δ-3-carene (5.3%), citronellal (4.9%) and geranial (2.2%) as main compounds [17]. Popova et al. [18] described a Bulgarian *Melissa* oil containing 18.5% citronellal, 15.2%, geraniol, 9.5% citronellol, 7.2% geranyl acetate and 5.9% geranial.

Greek lemon balm leaf oils with α-pinene, β-pinene, sabinene, β-caryophyllene, caryophyllene oxide and germacrene D as reported by Basta et al. [19] were therefore presumed to derive from subsp. *altissima*. A leaf oil from Jordan having as main compound caryophyllene oxide (43.6%) reportedly also contained considerable amounts of γ-muurolene (28.8%) [20]. In this case a confusion of this latter compound with germacrene D appears probable, as both components have similar retention behavior and fragmentation patterns in GC/MS. In some oils these citrus aroma monoterpenes occur along with comparable amounts of sesquiterpenes as in the case of a Moroccon leaf oil with 14.4% citronellal, 10.2% geranyl acetate, 5.2% nerylacetate, 11.0% caryophyllene oxide and 8.2% β-caryophyllene [21] but also in various lemon balm strains from Poland [22].

Besides genetic factors such as the existence of the two subspecies, the basis of this observed high variability remains complex: There is the experience of the present study that second MOFF leaf oils had a highly uniform composition with little varying neral and geranial contents in contrast to the oils from the first cut of the same plants. Similarly, plants cultivated in Poland had in two consecutive years nearly the same geranial (45%) and neral (33%) contents in their oils while the citronellal content varied (0.4–8.7%) [23]. A further experiment from Poland reported higher neral and geranial levels under higher insolation [22]. In pot experiments, soil water content hardly influenced essential oil composition [24]. On the other hand, various accessions cultivated on two different sites in Turkey clearly differed in their oil composition [25].

### 2.4. Rosmarinic Acid and Total Phenolics

Like other species from the Lamiaceae lemon balm is rich in polyphenols. Therefore, total phenolics have been estimated by the colorimetric Folin-Ciocalteu- assay and rosmarinic acid has been determined by HPLC in ethanolic extracts of the samples. Rosmarinic acid contents (Table 3) ranged from 26.3 to 90.1 mg/g in the leaves and from 14.1 to 38.3 mg/g in the stems. The Pharmacopoeia Europaea [26] prescribes at least 1% of rosmarinic acid in the dried leaves. This requirement was met by all leaf samples. MOFF leaves had more rosmarinic acid than those of MALT (*p* = 0.038) and rosmarinic acid was higher in the leaves from the first cut as compared to the second cut (*p* = 0.020). Furthermore, in MALT leaf oils rosmarinic acid was positively correlated (*N* = 25) with caryophyllene oxide (*R* = 0.423, *p* = 0.035) and negatively correlated with sabinene (*R* = −0.667, *p* < 0.001), α-pinene (*R* = −0.649, *p* < 0.001) and β-pinene (*R* = −0.634, *p* = 0.001). An overview of total phenolics content expressed as rosmarinic acid equivalents per g dry matter is given in Table 3.

Again, the leaves were richer in total phenolics than the stems. For the leaves the values ranged between 47.3 and 104.2 mg/g, but the mean contents of the accessions between cuts or the subspecies were not significantly different. For the stems total phenolic contents were between 26.7 and 57.6 mg/g with markedly lower contents in the MALT samples (*p* < 0.001). Total phenolics from 70% ethanolic lemon balm extracts were calculated as 69.5 to 76.4 mg/g gallic acid equivalents [27] and were thereby comparable to the contents observed in the present study. With 98.5 mg/g caffeic acid equivalents, lemon balm leaves from Greece were in the same range [28]. By relating the HPLC data with the colorimetric rosmarinic acid equivalent data the percentage of rosmarinic acid in the total phenolics was calculated. For the leaves the mean proportion of rosmarinic acid was between 73.8% and 85.8%. In stems this percentage was lower, ranging from 45.9% to 69.4%, so rosmarinic acid was the major polyphenolic compound. Infusions of various lemon balm leaves gave on average 21.9 mg/g rosmarinic acid [29]. A hydro-alcoholic extract of *Melissa* gave about 29.7 mg/g rosmarinic acid [30]. Carnat et al. [31] reported 4.1% rosmarinic acid in lemon balm leaves. In the chromatograms of the present study several further peaks were present besides rosmarinic acid but they were not further identified. Literature reports mention various polyphenolic compounds for lemon balm leaves and rosmarinic acid was often not a major compound. Lyophilized ethanolic extracts contained rutin as major flavonoid with 9.26%, followed by quercetin (4.13%), quercitrin (3.59%) and isoquercitrin (2.35%). Amongst phenolic acids, the most prevalent was caffeic acid with 6.42%, followed by ellagic (4.85%) and gallic acid (3.76%). In this study, rosmarinic acid was only a minor compound (2.2%) [32]. Another study reported sinapic and ferulic acid along with rosmarinic acid as main compounds [27]. Furthermore, extracts obtained with different techniques contained lithospermic acid, salvianolic acid and their derivatives [4,33].

### 2.5. Antioxidant Activity

Additionally, antioxidant parameters as DPPH radical scavenging activity and ferric reducing antioxidant power (FRAP) were measured in the extracts. The results in Trolox equivalents of the DPPH assay in Table 3 gave 45.7 to 154.0 mg/g for the leaves and 18.9 to 77.3 mg/g for the stems. In leaves this activity was lower in first cut than in the second (*p* < 0.001). MALT stems were lower in antioxidants than MOFF stems (*p* = 0.04). Similarly, the FRAP assay (Table 3) gave higher values for the leaves (72.1–190.9 mg/g Trolox equivalents) than for the stems (14.9–93.1 mg/g Trolox equivalents). In this case the stems of the second cut had the lower levels than those of the first (*p* = 0.021). When correlating rosmarinic acid, total phenolics, DPPH and FRAP antioxidant activity with each other in all cases highly significant correlations could be obtained. The correlation coefficients for the six possible combinations ranged between 0.901 and 0.941 with *N* = 72–75 and always *p* < 0.001. Accordingly, these four variables grouped closely together on the loading plot in PCA (Figure 3) away from essential oil compounds. All together the activities reported here are comparable to those reported earlier by Duda et al. [27] who found around 125 mg/g and 135 mg/g Trolox equivalents in the DPPH assay and FRAP assay, respectively, and by Skotti et al. [28] relating 158 mg/g Trolox equivalents.

## 3. Materials and Methods

### 3.1. Plant Material

Central European climate conditions allow cultivating lemon balm in permanent fields and harvesting the vegetative herb several times a year. Twenty-eight accessions of lemon balm were grown in field plots at the Leibniz Institute of Plant Genetics and Crop Plant Research (IPK) in Gatersleben, Germany (Table 4).

The plots were established in March 2008; a morphological evaluation was carried out in September 2009 where the following characters were recorded: plant height, length of the shoot in the lower plant part, internode length in the lower plant part, leaf length and leaf width on ten individuals from each accession. For the chemical analyses, the plants were harvested twice, in June and August 2013. The plant material was dried in a drying chamber with 18 °C and a relative humidity of 20% and then separated into leaves and stems.

### 3.2. Analyis of the Essential Oils

#### 3.2.1. Hydrodistillation

About 15 g of dried plant material were hydrodistilled with 400 mL double distilled water in a Clevenger type apparatus for 1.5 h. As the plant material contained low oil amounts, 1.0 mL *n*-hexane was added into the apparatus to collect the volatile oils. All accessions were distilled twice and therefore also analyzed twice. The collected volatile fractions were stored at −18 °C until further GC and GC-MS analysis. Prior to analysis cyclododecanone in hexane was added to the samples as an internal standard.

#### 3.2.2. Gas Chromatography/Mass Spectrometry (GC/MS) and Gas Chromatography/Flame Ionization Detection (GC/FID)

The analyses were carried out on an Agilent Technologies 7890A gas chromatograph equipped with a 5975 C quadrupole mass selective detector, a flame ionization detector (FID) and a CTC-PAL autosampler (Agilent Technolgies, Santa Clara, CA, USA). The separation was done on a 30 m × 0.25 mm fused silica column coated with 0.25 µm HP5-MS. The compounds eluting from the column were distributed with a Deans switch at equal proportions to the detector of the mass spectrometer (MSD) and FID. The temperature program of the oven was: isotherm at 50 °C for 1 min then increasing to 220 °C at a rate of 5 °C/min and increasing further to 280 °C at a rate of 15 °C/min. The split of the injector was set at 1:30. The injection volume was 1 µL. The total ion current (*m*/*z* 40 to 400) from the MSD was used to identify the compounds according their mass spectra and their retention indices [34,35,36,37]. The peak areas of the FID signal were used to calculate the percental composition of the oils without any correction. As a range of accessions had very low oil contents, so that the oil amount could not be read at the distillation unit, the oil content was estimated through the total peak area assuming the same response as the internal standard.

### 3.3. Analysis of Polyphenols and Antioxidant Activity

#### 3.3.1. Extraction

About 100 mg of the finely powdered leaves were extracted with 5 mL methanol for 30 min in an ultrasonic bath. The extracts were filtered and kept at −18 °C until further analysis. Before analysis leaf and stem extracts were diluted 1:5 and 1:3 with methanol, respectively.

#### 3.3.2. HPLC

The content of rosmarinic acid was measured according to Chizzola et al. [36] using a Waters HPLC system consisting of a 626 pump, a 600S controller, a 717plus autosampler, a column oven operated at 25 °C, and a 996 diode array detector (Waters S.A.S, Saint-Quentin, France). The separation was carried out on a Symmetry C18, 5.0 μm particle size, 4.6 × 150 mm column. The mobile phase used was 1% acetic acid/acetontrile 85:15 (solvent A) and methanol (solvent B). The analysis started with a solvent ratio of A/B of 9:1, and a linear gradient was performed to reach 100% B within 30 min. The flow rate was 1.0 mL/min and the injection volume, 20 μL.

The quantification of rosmarinic acid was done using the external standard method by preparing seven calibration standards ranging from 3.9 to 500 μg/mL and recording the calibration curve at 330 nm. A calibration line with the correlation coefficient *R*^2^ 0.997 could be established.

The following measurements of polyphenols and antioxidative activity were based on colorimetric reactions and were adapted to be measured with an iMark microplate reader (BioRad, Hercules, CA, USA). On a microplate four replicates of each sample or standard were carried out as described by Chizzola et al. [37].

#### 3.3.3. Total Phenolics

This fraction was assayed with the Folin-Ciocalteu reagent. In the wells of the microplate 10 µL extracts was added to 100 µL aqua dest. followed by 5 µL Folin-Ciocalteu reagent, 10 µL Na_2_CO_3_ (35% in aqua dest.) and again 125 µL aqua dest. After 1 h resting in the dark the plate is measured at 750 nm. To calibrate the color formation six concentration steps (ranging from 0.4 to 4.0 µg rosmarinic acid in 110 µL were taken instead the sample and the initial water volume. 

#### 3.3.4. DPPH-Test

Antioxidants react with the stable 2,2-diphenyl-1-picrylhydrazyl radical (DPPH) which is then decolorized. A portion of the sample (5–25 µL) made up to 100 µL methanol were incubated for 30 min in the dark with 100 µL DPPH reagent (0.015% in methanol) in the microplate wells. Increasing volumes (0–8 µL) of Trolox (0.62 mg/mL in ethanol) made up to 100 µL with methanol instead of the samples were used to obtain a calibration curve. A preparation consisting of 50 µL trolox, 50 µL aqua dest. and 100 µL DPPH reagent where the DPPH was completely decolorized was taken as blank and subtracted from all measurements. The decoloration was measured at 490 nm.

#### 3.3.5. FRAP Assay

The principle is based on the ability of antioxidants to reduce ferric (Fe^+++^) ions. The resulting ferrous ions (Fe^++^) form a deep blue complex with 2,4,6-tripyridyl-s-triazine (TPTZ) [38]. In the microplate wells 9 µL sample, 15 µL aqua dest. and 180 µL working reagent were mixed and measured after 5 min at 595 nm. The working reagent consisted of 25 mL acetic acid buffer (300 mmol/L), pH 3.6, 2.5 mL 10 mmol/L TPTZ in 40 mmol/L HCl and 2.5 mL FeCl_3_ solution (20 mmol/L). A calibration curve was generated using increasing amounts of trolox from 0.06 to 2.4 µg/well (7 steps) instead of the samples.

### 3.4. Statistical Analysis

The statistical analyses were done with the package IBM SPSS for Windows, version 22.0 (IBM Corporation, Armonk, NY, USA). A hierarchical cluster analysis (HCA) using the squared Euclidian distance with linkage between groups was carried out for each subspecies to group the accessions according to the composition of their leaf essential oils from both cuts, considering the percentages of 17 oil compounds. Furthermore, the complex interplay of major essential oil compounds was studied by principal component analysis (PCA). The percentages of 17 essential oil compounds, DPPH and FRAP as trolox-equivalents, total phenolics as rosmarinic acid equivalents and rosmarinic acid (mg/g DM) have been used as variables (see the legend of Figure 3). The leaf samples from both cuts were the cases. The software performed a Z-transformation before calculating the loadings of the variables and scores of the samples. Pearson correlation coefficients were calculated between selected essential oil components and rosmarinic acid. One-way analysis of variance was applied to evaluate differences in rosmarinic acid contents and antioxidant parameters.

## 4. Conclusions

Both lemon balm subspecies can clearly be differentiated by the composition of their essential oils. The MALT leaf oils showed higher variation than those of MOFF. Comparing the two cuts, the oil production displayed a remarkable plasticity. The oil composition of MOFF plants shifted from citronellal, geranial and neral in variable proportion in the first cut to geranial and neral in an approximate ratio of 1.47 and representing 80–90% of the respective oils. In contrast, MALT plants had oils with sesquiterpenes in the first cut that were augmented by sabinene and the pinenes in the second cut. In the leaves, the lowest and highest rosmarinic acid contents differed approximately by the factor three with a corresponding variability in total phenolics and antioxidant activity. This plasticity might be the base for the selection of lines and optimization of cultivation to obtain highly valuable plants high in rosmarinic acid and citrus-like aroma aldehydes.

## Figures and Tables

**Table 1:** Variability of morphological characters in lemon balm.

	**Plant Height (cm)**				**Shoot Length (cm)**			
	subsp. *officinalis*		subsp. *altissima*		subsp. *officinalis*		subsp. *altissima*	
Mean *	43.7	±14.6	54.0	±15.0	20.8	±4.5	28.4	±7.2
Min	25.4	*M1*	27.6	*M19*	11.2	*M1*	18.1	*M19*
Max	67.5	*M27*	88.1	*M20*	29.5	*M27*	42.4	*M22*
	**Internode Length, Lower Plant Part (cm)**				**Leaf Width (cm)**			
	subsp. *officinalis*		subsp. *altissima*		subsp. *officinalis*		subsp. *altissima*	
Mean	2.54	±0.58	3.83	±0.78	3.87	±0.54	4.21	±0.40
Min	1.37	*M1*	2.73	*M9*	2.82	*M4*	3.67	*M18*
Max	3.31	*M27*	4.91	*M20*	4.59	*M10*	5.09	*M9*
	**Leaf Length (cm)**				**Ratio Leaf Length/Width**			
	subsp. *officinalis*		subsp. *altissima*		subsp. *officinalis*		subsp. *altissima*	
Mean	4.38	±0.62	4.54	±0.49	1.138	±0.06	1.082	±0.05
Min	3.32	*M1*	3.99	*M19*	1.022	*M26*	1.001	*M19*
Max	5.53	*M7*	5.64	*M9*	1.251	*M7*	1.181	*M22*

* Mean ± standard deviation from 15 and 13 accessions of the subsp. *officinalis* and *altissima*, respectively.

**Table 2:** Essential oils in leaves and stems of lemon balm, *Melissa officinalis* ssp. *officinalis* (MOFF) and *M. officinalis* ssp. *altissima* (MALT), (µg/g, calculated from the FID signal).

		Leaves				Stems			
Cut		MOFF (*N* = 15)		MALT (*N* = 13)		MOFF (*N* = 5)		MALT (*N* = 5)	
1	Mean *	1278	±1294	130.9	±85.1	19.5	±11.2	7.6	±7.2
	Min	293	*M13*	59.1	*M14*	8.2	*M4*	0.1	*M21*
	Max	5083	*M1*	388.8	*M17*	36.5	*M27*	15.3	*M17*
2	Mean *	7650	±2325	1697	±822	486.6	±675.9	68.8	±25.0
	Min	4753	*M5*	909.2	*M24*	50.4	*M1*	38.4	*M12*
	Max	12669	*M26*	2759	*M22*	1611	*M4*	93.1	*M17*

* Mean ± standard deviation.

**Table 3:** Rosmarinic acid (mg/g DM), total phenolics (mg/g DM rosmarinic acid equivalents), DPPH (mg/g DM Trolox equivalents) and FRAP (mg/g DM Trolox equivalents) in leaves and stems of lemon balm subspecies *officinalis* (MOFF) and subspecies *altissima* (MALT).

		Leaves				Stems			
Cut		MOFF (*N* = 15)		MALT (*N* = 13)		MOFF (*N* = 5)		MALT (*N* = 5)	
	RA								
1	Mean	70.0	±10.8	65.9	±13.2	37.2	±4.4	26.8	±6.2
	Min	52.1	*M16*	38.4	*M22*	32.1	*M5*	16.6	*M22*
	Max	85.7	*M1*	80.5	*M17*	42.2	*M29*	33.5	*M20*
2	Mean	64.8	±11.0	52.9	±16.3	24.4	±9.3	22.3	±9.3
	Min	53.2	*M10*	26.3	*M21*	16.0	*M16*	14.1	*M20*
	Max	90.1	*M13*	75.5	*M14*	34.4	*M6*	38.3	*M24*
	Total Phenolics								
1	Mean	81.9	12.2	81.0	8.4	53.7	7.3	37.1	6.0
	Min	62.2	*M11*	63.7	*M22*	47.4	*M16*	26.7	*M22*
	Max	101.7	*M29*	90.7	*M20*	62.3	*M25*	42.0	*M20*
2	Mean	79.5	11.5	70.7	16.2	53.8	4.1	34.1	4.1
	Min	64.6	*M27*	47.3	*M21*	47.1	*M5*	30.4	*M22*
	Max	100.7	*M13*	104.2	*M14*	57.6	*M6*	40.4	*M24*
	DPPH								
1	Mean	124.4	±18.8	120.7	±14.2	58.4	±13.5	31.5	±7.3
	Min	91.6	*M11*	92.2	*M22*	45.7	*M16*	18.9	*M22*
	Max	154.0	*M1*	138.1	*M17*	77.3	*M5*	37.2	*M20*
2	Mean	104.9	±22.8	85.4	±25.3	56.8	±16.9	34.4	±27.3
	Min	61.4	*M27*	43.9	*M28*	36.0	*M5*	11.3	*M22*
	Max	138.2	*M1*	137.4	*M14*	77.4	*M25*	81.2	*M24*
	FRAP								
1	Mean	132.0	24.8	134.7	17.8	87.3	9.7	63.5	11.3
	Min	91.4	*M16*	108.1	*M22*	74.4	*M29*	44.5	*M22*
	Max	190.9	*M29*	166.0	*M20*	101.7	*M25*	72.8	*M17*
2	Mean	134.9	17.8	111.7	27.1	66.7	20.1	32.1	26.0
	Min	103.5	*M16*	72.1	*M21*	41.3	*M5*	14.9	*M17*
	Max	159.8	*M6*	160.0	*M14*	93.1	*M25*	77.9	*M24*

**Table 4:** List of *Melissa officinalis* accessions from IPK.

Nr	Accession	Subspecies	Variety	Date *	Country of Origin
*M1*	MELI 1	*officinalis*		1950	
*M2*	MELI 2	*officinalis*		1950	
*M4*	MELI 4	*officinalis*		1976	Germany
*M5*	MELI 5	*officinalis*	Erfurter Aufrechte	1975	Germany
*M6*	MELI 6	*officinalis*		1975	Germany
*M7*	MELI 7	*officinalis*	Citra	1978	
*M8*	MELI 8	*officinalis*		1986	Georgia
*M9*	MELI 9	*officinalis*		1986	France
*M10*	MELI 10	*officinalis*		1986	France
*M11*	MELI 11	*officinalis*	Cedronella	1988	Italy
*M12*	MELI 12	*altissima*		1988	Italy
*M13*	MELI 13	*officinalis*		1989	Georgia
*M14*	MELI 14	*altissima*		1990	Italy
*M15*	MELI 15	*altissima*		1990	Italy
*M16*	MELI 16	*officinalis*		1994	
*M17*	MELI 17	*altissima*		1994	Greece
*M18*	MELI 18	*altissima*		1994	
*M19*	MELI 19	*altissima*		1997	Italy
*M20*	MELI 20	*altissima*		1997	Italy
*M21*	MELI 21	*altissima*		1994	Albania
*M22*	MELI 22	*altissima*		1994	Turkey
*M23*	MELI 23	*altissima*		1998	Italy
*M24*	MELI 24	*altissima*		1998	Italy
*M25*	MELI 25	*officinalis*		2002	
*M26*	MELI 26	*officinalis*		2002	Armenia
*M27*	MELI 27	*officinalis*		2003	Italy
*M28*	MELI 28	*altissima*		2003	Italy
*M29*	MELI 29	*officinalis*		2002	

For further details see: https://gbis.ipk-gatersleben.de/GBIS_I/ergebnisliste.jsf;jsessionid=FVKiZMR28paA15 qWaQcWOiYCMmB3iZ8qySJuNW3GErIkuzlsqhKx!363099909!1514553001078 accessed: 29.12.2017; * refers to year of acquisition of the respective accession.

## Supplementary Materials

The following are available online.
