# Supplementary material for: Biodiversity within Melissa officinalis: Variability of Bioactive Compounds in a Cultivated Collection
Source: Molecules. 2018 Jan 31;23(2):294. doi: 10.3390/molecules23020294 (PMC6017880; doi:10.3390/molecules23020294)
Supplement: Supplementary file 1 [file molecules-23-00294-s001.pdf]

Supporting Information to

Biodiversity within *Melissa officinalis*: Variabiliy of Bioactive Compounds in a Cultivated Collection

Table S1: Oil yield in leaves and stems of lemon balm, *Melissa officinalis* ssp. *officinalis* (MOFF) and *M. officinalis* ssp. *altissima* (MALT), (µg/g, calculated from the FID signal)

Table S2: Composition of the leaf essential oils from *Melissa officinalis* (% of total peak area on a apolar HP5-MS column, calculated from the FID signal)

Table S3: Composition of the stem essential oils from *Melissa officinalis* (% of total peak area on a apolar HP5-MS column, calculated from the FID signal)

Figure S1: *Melissa officinalis*: composition of the leaf essential oils from the 1st cut

Figure S2: *Melissa officinalis*: composition of the leaf essential oils from the 2nd cut

Figure S3: *Melissa officinalis*: composition of the stem essential oils from the 1st cut

Figure S4: *Melissa officinalis*: composition of the stem essential oils from the 2nd cut

Table S1: Oil yield in leaves and stems of lemon balm, *Melissa officinalis* ssp. *officinalis* (MOFF) and *M. officinalis* ssp. *altissima* (MALT), (μg/g, calculated from the FID signal)

Accession	1st cut	2nd cut	1st cut	2nd cut
	Leaves		Stems	
MOFF				
M1	5083	7180	14.1	50.4
M2	1150	5652		
M4	3210	12245	8.2	1612
M5	845	4753		
M6	1937	6512		
M7	414	6745		
M8	893	8995		
M10	1539	6311	14.2	82.3
M11	667	6902		
M13	293	5234		
M16	843	6158		
M25	554	8968		
M26	782	12669	24.6	634
M27	335	8358	36.5	54.6
M29	627	8062		
MALT				
M9	807	-		
M12	167	2731	8.0	38.4
M14	59	1634		
M15	76	1033		
M17	389	3212	14.0	93.1
M18	139	1313		
M19	100	996		
M20	117	1081	15.3	85.7
M21	164	2220	0.1	45.4
M22	128	2759	0.4	81.3
M23	84	1531		
M24	68	909		
M28	129	947		

Table S2: Composition of the leaf essential oils from *Melissa officinalis* (% of total peak area on a apolar HP5-MS column, calculated from the FID signal)

		subsp. *officinalis* MOFF				subsp. *altissima* MALT			
**Compound**	**RI**	1st cut		2nd cut		1st cut		2nd cut	
		mean*	*SD*	mean*	*SD*	mean*	*SD*	mean*	*SD*
trans-2-Hexenal	**856**	<0.05	*<0.1*	0.1	*0.1*	0.2	*0.5*	<0.05	
α-Thujene	**933**			<0.05		0.1	*0.2*	0.8	*0.4*
α-Pinene	**942**			<0.05		0.5	*1.3*	5.0	*2.9*
Camphene	**956**							0.1	*0.1*
1-Octen-3-ol	**981**	0.3	*0.2*	0.2	*0.1*	0.3	*0.8*		
Sabinene	**982**			0.1	*0.4*	1.6	*3.6*	14.7	*8.3*
β-Pinene	**986**			0.1	*0.4*	1.5	*3.1*	11.9	*5.8*
6-Methyl-5-heptene-2-one	**989**	0.3	*0.1*	0.5	*0.2*	0.5	*1.1*		
Myrcene	**994**	<0.05		0.2	*<0.1*	<0.05		0.4	*0.2*
α-Terpinen	**1022**					0.1	*0.2*	0.5	*0.3*
p-Cymene	**1031**					<0.05		0.1	*0.1*
Limonene }<br>β-Phellandrene }	**1036**<br>**1036**					0.1	*0.3*	1.3	*0.6*
1,8-Cineol	**1038**							0.3	*0.1*
Z-β-Ocimene	**1042**	<0.05		<0.05		0.1	*0.3*	0.4	*0.1*
E-β-Ocimene	**1053**	0.6	*0.4*	0.2	*0.1*	0.3	*0.5*	1.4	*0.5*
2,6-Dimethyl hept-5-en-1-al	**1059**	0.2	*0.1*						
γ-Terpinene	**1065**					0.2	*0.5*	1.3	*0.5*
Terpinolene	**1093**			<0.05		<0.05		0.3	*0.1*
Linalool	**1102**	0.7	*0.3*	0.3	*0.2*	0.1	*0.2*	0.1	*0.2*
cis-Roseoxide	**1115**	0.1	*0.1*						
trans-Roseoxide	**1133**	0.1	*0.1*						
α-Campholenaldehyd	**1133**	<0.05				<0.05		0.1	*<0.1*
Photocitral	**1147**	0.1	*0.1*	0.1	*<0.1*				
trans-Pinocarveol	**1148**					0.2	*0.4*	0.3	*0.1*
exo-Isocitral	**1150**	0.2	*0.1*	0.3	*<0.1*				
Isopulegol	**1154**	0.1	*0.1*			<0.05			
Citronellal	**1160**	20.6	*8.4*	0.8	*0.3*	0.5	*1.0*	0.3	*0.2*
iso-Isopulegol	**1166**	0.1	*0.1*						
Z-Isocitral	**1170**	1.3	*0.3*	1.4	*0.2*				
Pinocarvone	**1172**					0.1	*0.2*	0.2	*0.1*
Rosefuran epoxide	**1180**	<0.05		0.1	*0.1*				
Terpinen-4-ol	**1185**							1.2	*0.5*
E-Isocitral	**1187**	1.8	*0.4*	2.0	*0.2*	0.2	*0.6*		
α-Terpineol	**1196**					0.1	*0.5*	0.1	*0.1*
Myrtenal	**1204**					0.2	*0.6*	0.4	*0.1*
Citronellol	**1235**	0.2	*0.2*	<0.05					
Neral	**1253**	19.9	*5.1*	35.0	*1.6*	0.4	*1.0*	0.4	*0.5*
Geranyl formate	**1262**	0.1	*0.1*	0.1	*0.1*				

Table S2: continued

		subsp. *officinalis*				subsp. *altissima*			
**Compound**	**RI**	1st cut		2nd cut		1st cut		2nd cut	
		mean*	*SD*	mean*	*SD*	Mean*	*SD*	Mean*	*SD*
Methyl citronellate	**1265**	1.1	*0.5*	<0.05					
Geranial	**1283**	30.8	*7.0*	51.3	*2.6*	0.7	*1.8*	0.6	*0.8*
Methyl geranate	**1328**	0.3	*0.1*	0.2	*0.2*				
Geranic acid	**1357**	<0.05		0.1	*0.4*	0.2	*0.7*		
α-Copaene	**1386**	0.5	*0.4*	1.0	*0.4*	3.5	*2.4*	1.7	*1.2*
β-Bourbonene	**1397**	<0.05				0.7	*0.4*	0.2	*0.1*
β-Elemene	**1400**	0.2	*0.3*			1.3	*0.7*	1.0	*0.4*
β-Caryophyllene	**1432**	4.0	*2.8*	3.1	*1.1*	9.9	*3.0*	17.2	*6.9*
β-Gurjunene	**1442**							0.1	*<0.1*
E-β-Farnesene	**1462**	0.1	*0.1*	<0.05		<0.05			
α-Humulene	**1469**	0.3	*0.2*	0.2	*0.1*	0.6	*0.4*	1.1	*0.4*
9-*epi*-*E*-Caryophyllene	**1477**	<0.05		<0.05		0.6	*0.6*	2.5	*1.6*
α-Amorphene	**1489**							0.2	*0.1*
Germacrene D	**1492**	1.2	*1.9*	0.2	*0.2*	15.4	*12.5*	19.6	*7.1*
Z,E-α-Farnesene	**1498**	0.2	*0.5*	<0.05					
γ-Amorphene	**1505**							0.2	*0.1*
Bicyclogermacrene	**1507**							0.2	*0.4*
E,E-α-Farnesene	**1513**	0.1	*0.2*	<0.05		0.5	*0.5*	0.5	*0.4*
γ-Cadinene	**1529**	<0.05				0.3	*0.3*	0.2	*0.1*
δ-Cadinene	**1536**	0.3	*0.3*	<0.05		1.2	*0.6*	0.8	*0.7*
MW 220**	**1569**	0.5	*0,2*	<0.05		2.8	*1.2*	0.7	*0.4*
Spathulenol	**1592**							0.1	*0.1*
Caryophyllenoxide	**1600**	9.2	*3.6*	1.3	*0.6*	36.6	*12.9*	7.1	*3.0*
MW 220**	**1627**	0.6	*0.2*	0.1	*0.1*	1.4	*0.9*	0.4	*0.1*
Caryophylla-4(12),8(13)-dien-5ol	**1654**	0.1				0.5	*0.5*	0.2	*0.2*
α-Cadinol	**1670**	0.3	*0.3*	<0.05	*0.1*	0.8	*1.0*	0.9	*0.7*
MW 220**	**1686**	0.5	*0.2*			3.2	*0.8*	0.5	*0.2*
MW 220**	**1702**	0.1	*0.1*			0.4	*0.6*	0.3	*0.1*
2E,6E-Farnesal	**1750**							0.1	*0.1*
Hexahydrofarnesylacetone	**1849**	0.1	*0.1*			0.1	*0.2*	0.1	*0.1*
Hexadecanoic acid	**1960**	0.6	*0.3*	<0.05		2.6	*2.1*	0.1	*0.1*

* Mean of the accessions and standard deviation (SD), n=15 for MOFF and n=13 for MALT, ** oxidised sesquiterpene with molar mass 220

Table S3: Composition of the stem essential oils from *Melissa officinalis* (% of total peak area on a apolar HP5-MS column, calculated from the FID signal)

		subsp. *officinalis* MOFF				subsp. *altissima* MALT			
**Compound**	**RI**	1st cut		2nd cut		1st cut		2nd cut	
		mean*	*SD*	mean*	*SD*	mean*	*SD*	mean*	*SD*
α-Thujene	**933**							0.5	*0.1*
α-Pinene	**942**			<0.1	*0.1*	0.5	*1.1*	2.0	*1.8*
Sabinene	**982**			0.1	*0.2*	0.7	*1.5*	5.3	*3.9*
β-Pinene	**986**			0.1	*0.2*	1.0	*2.3*	5.7	*3.9*
6-Methyl-5-heptene-2-one	**989**			0.1	*0.2*				
Myrcene	**994**	0.1	*0.2*	0.1	*0.1*			0.1	*0.2*
α-Terpinen	**1022**							0.5	*0.5*
p-Cymene	**1031**							<0.1	*0.1*
Limonene }	**1036**							0.4	*0.4*
β-Phellandrene }	**1036**								
1,8-Cineol	**1038**							<0.05	*0.1*
Z-β-Ocimene	**1042**							<0.05	*0.1*
E-β-Ocimene	**1053**	0.1	*0.2*	<0.05	*<0.05*			0.3	*0.2*
γ-Terpinene	**1065**					0.4	*1.0*	1.4	*0.9*
Terpinolene	**1093**							0.3	*0.3*
Linalool	**1102**			0.1	*0.1*				
trans-Roseoxide	**1133**	0.7	*0.8*						
exo-Isocitral	**1150**			0.2	*0.1*				
Citronellal	**1160**	9.8	*7.1*	1.1	*0.9*				
Z-Isocitral	**1170**			1.1	*0.3*				
Pinocarvone	**1172**							0.1	*0.1*
Rosefuran epoxide	**1180**	1.6	*3.6*	1.3	*2.7*	1.5	*2.3*		
Terpinen-4-ol	**1185**							0.4	*0.4*
Myrtenal	**1204**							0.1	*0.1*
Neral	**1253**	8.0	*2.7*	23.6	*6.5*			0.1	*0.3*
Geranial	**1283**	14.5	*5.5*	44.7	*11.0*			0.2	*0.5*
Methyl geranate	**1328**	0.1	*0.2*	0.5	*0.3*				
α-Copaene	**1386**	11.3	*4.2*	6.7	*1.7*	2.1	*3.5*	1.7	*2.6*
β-Bourbonene	**1397**			<0.05	*0.1*	0.5	*0.7*	0.7	*0.4*
β-Elemene	**1400**	0.1	*0.3*	0.1	*0.2*	0.6	*0.9*	1.4	*0.2*
β-Caryophyllene	**1432**	16.3	*4.0*	6.9	*5.0*	7.8	*7.7*	19.0	*9.0*
β-Gurjunene	**1442**							0.2	*0.1*
α-Humulene	**1469**	0.6	*0.5*	0.4	*0.3*	0.2	*0.5*	1.2	*0.6*
9-*epi*-*E*-Caryophyllene	**1477**			0.2	*0.4*			0.8	*1.0*
α-Amorphene	**1489**							0.2	*0.2*
Germacrene D	**1492**	1.9	*0.9*	1.9	*3.3*	49.6	*34.4*	31.6	*8.2*
Bicyclogermacrene	**1507**							0.9	*0.6*
δ-Cadinene	**1536**	0.2	*0.4*	0.1	*0.2*	0.4	*0.5*	0.7	*0.1*
MW 220**	**1569**	0.7	*0.9*						
Spathulenol	**1592**					1.3	*1.2*		

Table S3: continued

		subsp. *officinalis* MOFF				subsp. *altissima* MALT			
**Compound**	**RI**	1st cut		2nd cut		1st cut		2nd cut	
		mean*	*SD*	mean*	*SD*	mean*	*SD*	mean*	*SD*
Caryophyllenoxide	**1600**	24.3	*6.4*	4.6	*2.8*	26.6	*18.3*	14.8	*4.3*
Caryophylla-4(12),8(13)-dien-5ol	**1654**			0.4	*0.3*			0.9	*0.8*
α-Cadinol	**1670**			0.3	*0.4*	0.2	*0.5*	0.8	*0.3*
MW 220**	**1702**			0.1	*0.3*	0.3	*0.7*	0.9	*0.9*
Hexahydrofarnesylacetone	**1849**	1.1	*0.7*	0.3	*0.2*				
Hexadecanoic acid	**1960**	0.4	*1.0*						

* Mean of the accessions and standard deviation (SD), n = 5, ** oxidised sesquiterpene with molar mass 220

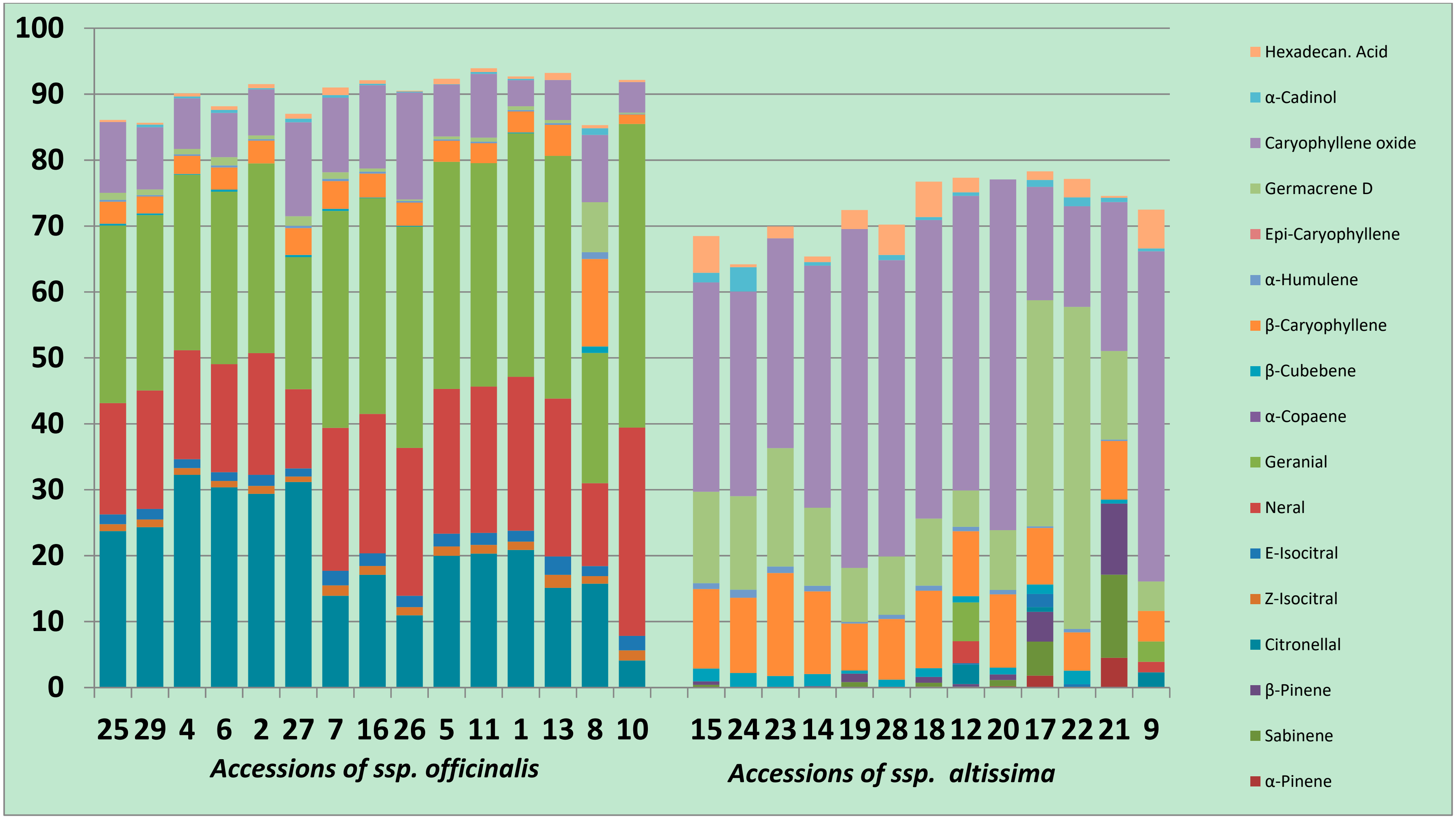

Figure S1: ***Melissa officinalis*****: composition of the leaf essential oils from the 1st cut**

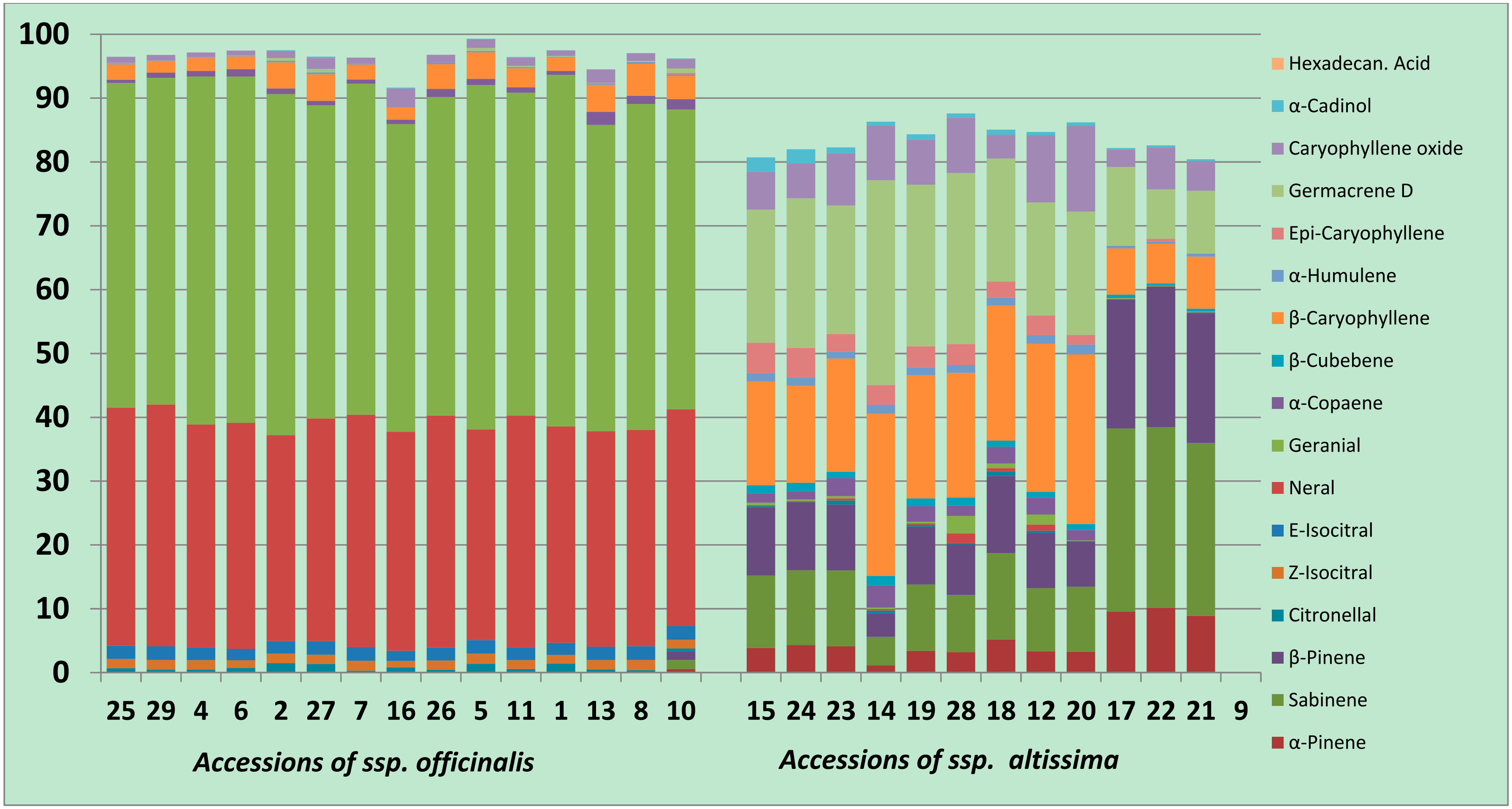

Figure S2: ***Melissa officinalis*****: composition of the leaf essential oils from the 2nd cut**

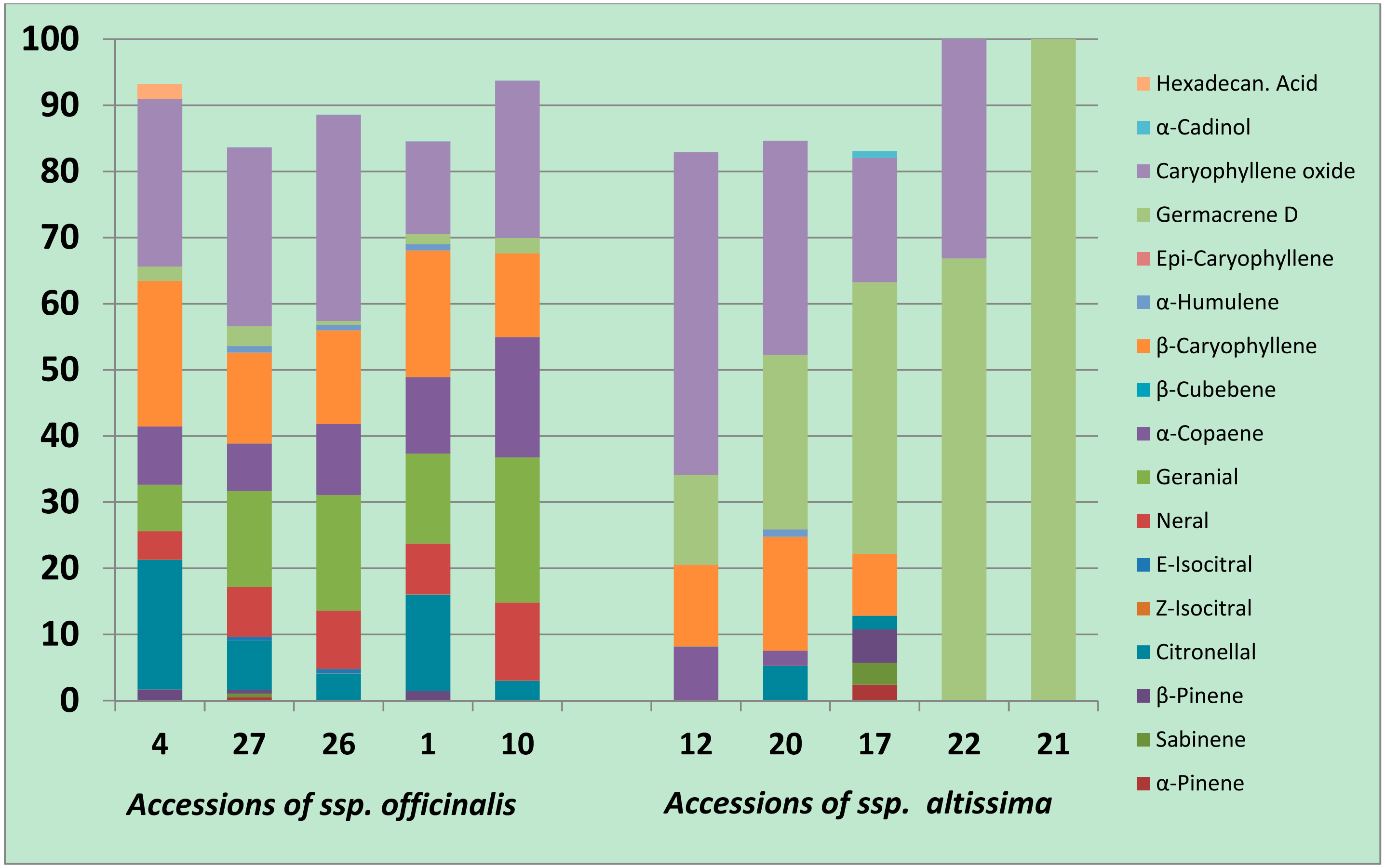

Figure S3: ***Melissa officinalis*****: composition of the stem essential oils from the 1st cut**

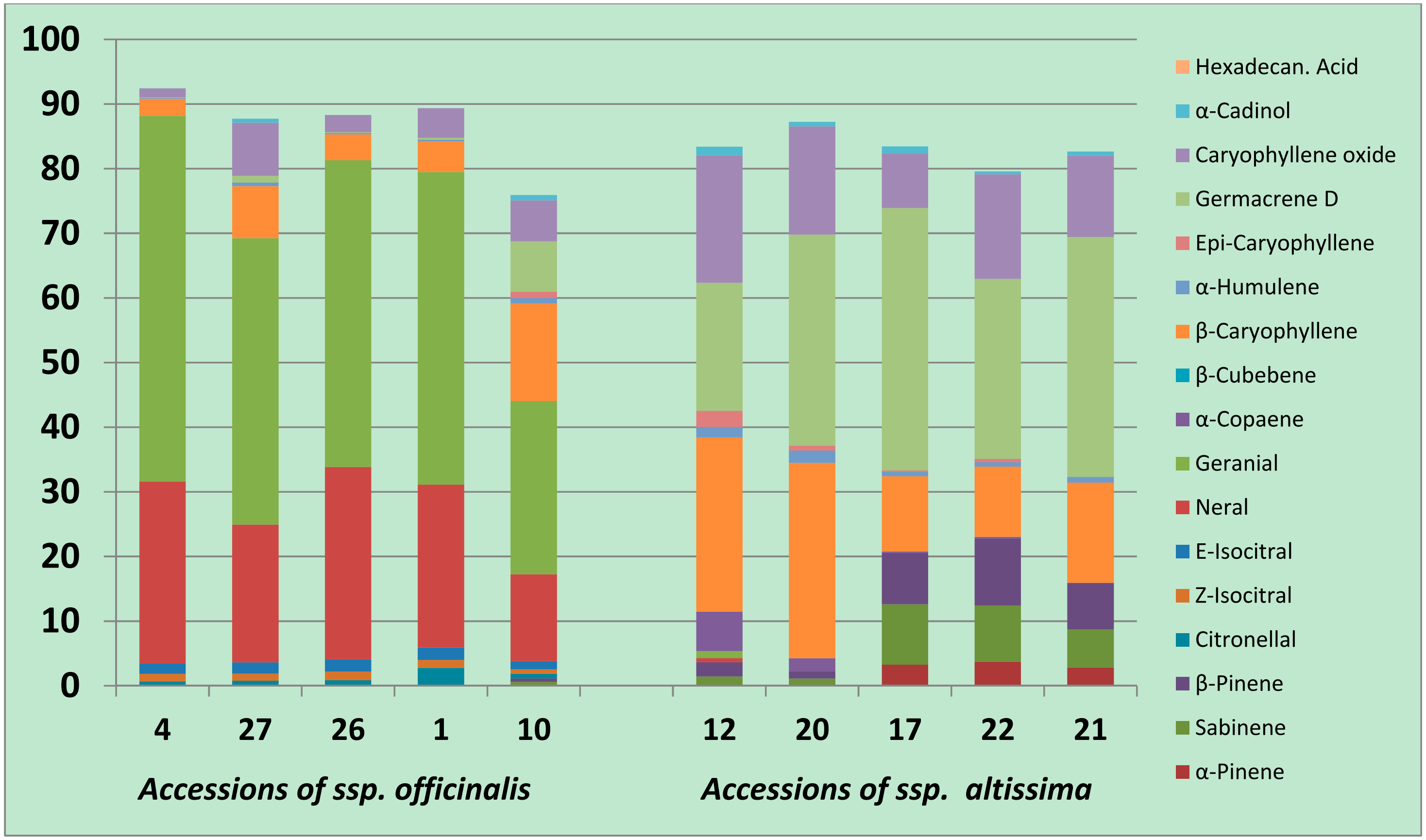

Figure S4: ***Melissa officinalis*****: composition of the stem essential oils from the 2nd cut**
